# Phase I Trial to Evaluate the Tolerance, Pharmacokinetics and Efficacy of the Broad-Spectrum ErbB Family Inhibitor Larotinib Mesylate in Patients With Advanced Solid Tumors

**DOI:** 10.3389/fphar.2021.636324

**Published:** 2021-02-18

**Authors:** Jingrui Liu, Hong Zhang, Xiaoxue Zhu, Hong Chen, Xiaojiao Li, Yanhua Ding

**Affiliations:** Phase I Clinical Trial Unit, The First Hospital of Jilin University, Jilin, China

**Keywords:** larotinib, phase I, dose escalation, ErbB family blocker, pharmacokinetics

## Abstract

**Background:** The presented phase I, first-in-human study evaluated the tolerance, pharmacokinetics, and preliminary efficacy of larotinib mesylate in patients with advanced solid tumors.

**Methods:** Cancer patients were assigned to receive larotinib mesylate at 50–400 mg dose levels until disease progression or intolerance. Dose-limiting toxicities were assessed during Cycles 0 and 1. Pharmacokinetic evaluations were performed after the first dose and at steady-state.

**Results:** Twenty-five patients with solid tumors were enrolled in the dose-escalation study. No DLTs were observed. Acne-like rash (68.0%), diarrhea (48.0%), paronychia (48.0%), and anemia (48.0%) were the most reported treatment-related adverse events. No clear linear pharmacokinetic characteristic could be drawn, and obvious accumulation was observed. Two patients with non-small cell lung cancer experienced a partial response, and 15 patients had stable disease after treatment.

**Conclusion:** Continuous oral administration of larotinib mesylate at 50–400 mg daily demonstrated a favorable safety profile, and anti-tumor activity was observed in patients with advanced solid tumors.

## Introduction

The ErbB family of tyrosine kinase receptors is composed of four members—human epidermal growth factor receptor 1 (HER1, also known as EGFR), HER2, HER3, and HER4, as well as their ligands. These receptors participate in the Ras/Raf/mitogen-activated protein kinase (MAPK), phosphatidylinositol-3-kinase (PI3K)/AKT, Janus kinase (JAK)/signal transducer and activator of transcription (STAT), and protein kinase C (PKC) signaling pathways (Hynes and Lane, 2005; Yarden and Pines, 2012). They play important roles in cell growth, differentiation, migration, and apoptosis (Yarden and Pines, 2012; Bahleda et al., 2015). Dysregulation of ErbB family receptors has been observed in many types of solid tumors. For this reason, ErbB family receptors, particularly EGFR and HER2, have been developed as targets for many anticancer drugs (Baselga, 2002; Normanno et al., 2005; Iivanainen and Elenius, 2010). To date, several small molecule EGFR tyrosine kinase inhibitors (TKIs) have shown clinical benefit in clinical trials and been approved for the treatment of malignancies, with especially dramatic improvements in the therapeutic outcomes in non-small cell lung cancer (NSCLC). The first-generation, reversible EGFR-TKIs include gefitinib and erlotinib (Mok et al., 2009; Maemondo et al., 2010; Mitsudomi et al., 2010; Zhou et al., 2011; Rosell et al., 2012; Wu et al., 2017), and the second-generation irreversible EGFR-TKIs include afatinib and dacomitinib (Yap et al., 2010; Bahleda et al., 2015; Park et al., 2016). The antitumor efficacy of these agents is severely impaired in most patients by the emergence of acquired resistance within 10–12 months (Sequist et al., 2013; Russo et al., 2017; Solassol et al., 2019). The acquired missense mutation in exon 20 of EGFR (T790M) has been identified as the most common causative factor in more than half of patients (Westover et al., 2018). The third-generation EGFR-TKI os imertinib has been developed to overcome the acquired T790M mutation and has been approved as a first-line drug for treating advanced EGFR-mutated NSCLC, but tumoral clone resistance often occurs after 6–17 months treatment (Wang et al., 2016; Mok et al., 2017; Tan et al., 2018; Solassol et al., 2019).

**FIGURE 1:**
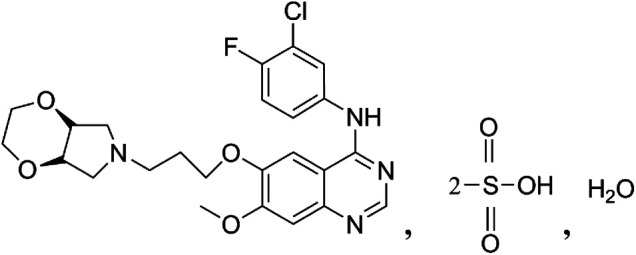
Structure of larotinib mesylate.

Larotinib mesylate (Figure 1) is a novel, potent broad-spectrum TKI with EGFR as the main target with a 50% inhibitory concentration (IC_50_) of 0.6 nM (data not published). It has shown distinct mechanisms of action from other first-generation and second-generation EGFR inhibitors on the market in that multiple EGFR subtypes were found to be potently inhibited in a larotinib mesylate *in vitro* study, including wild-type EGFR (IC_50_ = 0.611 nM, 3-fold and 37-fold greater inhibitory activity than erlotinib and gefitinib, respectively) (Moyer et al., 1997; Baselga and Averbuch, 2000), EGFR with the L858R mutation (IC_50_ = 0.563 nM), EGFR with the L861Q mutation (IC_50_ = 0.423 nM), and EGFR with a deletion in the exon 19. With respect to the most common resistance mechanism against first-generation agents, the acquired missense mutation in exon 20 of EGFR (T790M), larotinib mesylate also exhibits moderate inhibitory activity (IC50 = 45.2 nM). In addition, larotinib mesylate also inhibits the ErbB family receptors HER2 (IC50 = 253 nM) and HER4 (IC50 = 84 nM) as well as other kinases such as receptor-interacting serine/threonine-protein kinase 2 (RIPK2, IC50 = 26.6 nM), interleukin-1 receptor-associated kinase 1 (IRAK1, IC50 = 167 nM), Bruton’s tyrosine kinase (BTK, IC50 = 196 nM), B-lymphoid tyrosine kinase (BLK, IC50 = 102 nM), and more (data not published). We hypothesized that the characteristics of larotinib mesylate that facilitate simultaneous inhibition of multiple kinases may improve the tumor inhibiting effect of the drug and decrease the emergence of resistance to larotinib mesylate in solid tumor cells.

Animal toxicology studies of larotinib mesylate were conducted in both Sprague–Dawley rats and beagle dogs. After daily administration of larotinib mesylate, the study results showed that the no observed adverse effect level (NOAEL) was 10 mg/kg in Sprague–Dawley rats and the lowest observed adverse effect level (LOAEL) was 5 mg/kg in beagle dogs. The maximal tolerable doses (MTDs) were 20 and 25 mg/kg in Sprague–Dawley rats and beagle dogs, respectively. Furthermore, a preclinical pharmacodynamic study conducted using tumor-bearing mice as a model showed dose-dependent antitumor results and a tumor-inhibiting rate exceeding 60% with a larotinib mesylate dose of 18 mg/kg (data not published).

According to the guidance for clinical trials of antitumor agents published by the Center for Drug Evaluation and Research in China, for cytotoxic antitumor agents, the initial dose in a phase I study can be calculated as 1/10 of the MTD in rodents or 1/6 of the MTD in non-rodent animals (CFDA, 2012). For non-cytotoxic antitumor agents, because of their relatively lower toxicity, the initial dose can be set higher. According to the reported preclinical results for larotinib mesylate, the MTD in Sprague–Dawley rats translated to a human equivalent dose of 192 mg/d according to body surface area (calculated by 60 kg body weight), for which 1/10 is 19.2 mg/d (FDA, 2005). Additionally, from the MTD in beagle dogs, the human equivalent dose was 810 mg/d, 1/6 of which is 135 mg/d. As for the effective dose of 18 mg/kg in tumor-bearing mice, it was translated to a human equivalent effective dose of 86.4 mg/d according to body surface area. From the results of *in vitro* metabolism test in liver microsome of different species, the similar metabolic patterns were observed between human and beagle dogs. It was considered that dog was the more related species to human than rat. And in the clinical trial of similar drugs erlotinib and gefitinib, the incidence of adverse events (AEs) were low in the low-dose groups (before 100 or 150 mg). Also as a well researched target, the main EGFR-related adverse events (AEs) were controllable skin or mucosal damages and gastrointestinal reactions. Taking the above results for the toxicology, preclinical pharmacology and pharmacodynamics, and drug specifications (10, 50 and 100 mg) of larotinib mesylate into account, we calculated a recommended starting dose of 50 mg larotinib mesylate for a phase I, first-in-human clinical study, lower than human equivalent 1/6 MTD translated from beagle dogs and near the human equivalent effective dose translated from tumor-bearing mice. For the highest dose design, the human equivalent MTDs were 192 and 810 mg translated from Sprague–Dawley rats and beagle dogs, respectively. Combined with the results of equivalent MTDs and equivalent effective dose, 400 mg was designed as the highest dose level in the study, exceeding the translated effective dose level and between the predicted MTDs for Sprague–Dawley rats and beagle dogs. Here we present this phase I, dose-escalation trial evaluating the tolerance, pharmacokinetics, and preliminary efficacy of larotinib mesylate at doses of 50–400 mg in patients with advanced solid tumors.

## Material and Methods

### Patients

Patients with advanced solid tumors were screened and enrolled in the study conducted at the First Hospital of Jilin University, China. The eligibility criteria were: pathologically or cytologically confirmed advanced solid tumor that had failed to respond to or was not suitable for standard therapeutic regimens; age range, 18–75 years with a body mass index of 18–30 kg/m^2^; at least one measurable lesion per the Response Evaluation Criteria In Solid Tumors (RECIST) (version 1.1) (Eisenhauer et al., 2009); at least a 3-months life expectancy, and an Eastern Cooperative Oncology Group (ECOG) performance score of 0–2; and remission of previous treatment-related adverse events (AEs) of Grade ≤1 according to the National Cancer Institute Common Terminology Criteria for Adverse Events (NCI CTCAE version 4.0). The exclusion criteria were: treatment with any other anti-tumor therapy within 4weeks before study entry; prior Grade ≥3 adverse reactions related to an EGFR TKI; symptomatic central nervous system metastasis; clinically significant arrhythmia or left ventricular ejection fraction (LVEF <45%); accompanying disease affecting gastrointestinal absorption; hepatitis B virus (HBV), hepatitis virus (HCV) or HIV infection; pregnancy or breastfeeding; and any other uncontrolled disease of the respiratory, circulatory, endocrine, or urinary systems. The study was approved by the ethics committee of the First Hospital of Jilin University and conducted under the guidelines of the Declaration of Helsinki and the International Conference on Harmonization Good Clinical Practice Guidelines. Written informed consent was obtained from all patients or their legal representatives prior to their enrollment.

### Study Design

**FIGURE 2:**
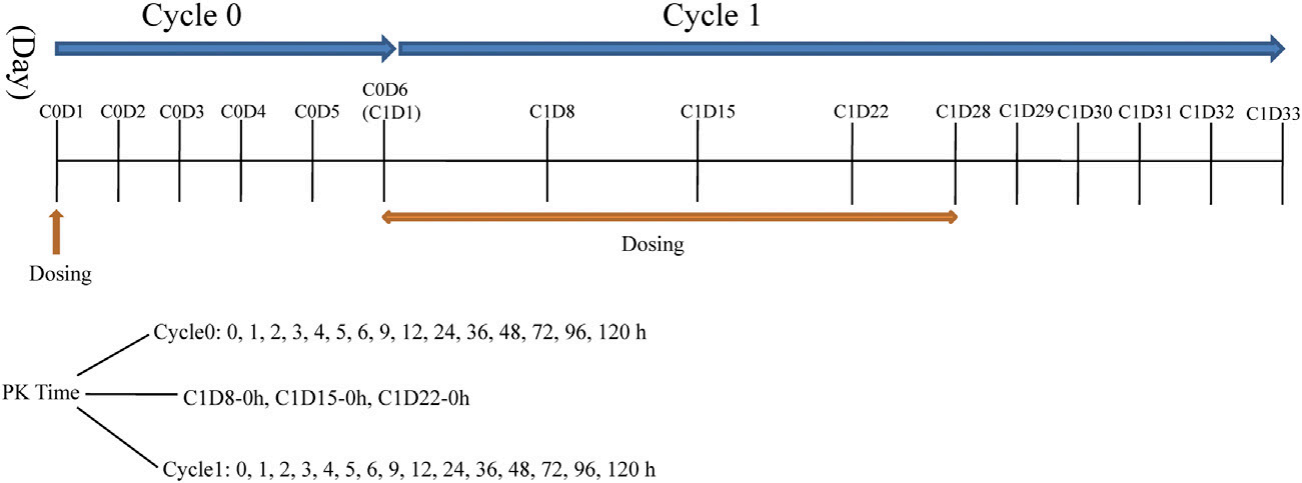
The dosing schedule and sampling time-points in Cycle 0 and 1.

This was a phase I, single-center, open-label, dose-escalation study conducted at First Hospital of Jilin University, China (registration no.: ChiCTR-OPC-15007153 registered at http://www.chictr.org.cn). Larotinib mesylate hard capsules were produced and supplied by Sunshine Lake Pharma Co., Ltd (Guangdong Province, China) in different unit doses of 10, 50, and 150 mg under GMP condition. Seven dose levels were designed in the study: 50, 100, 150, 220, 300, 350, and 400 mg with administration stages labeled cycle 0, cycle 1, and subsequent treatment cycles. Patients received an oral single dose of larotinib mesylate after fasting during a 6-days cycle 0 to evaluate the tolerance and pharmacokinetic characteristics. After cycle 0, larotinib mesylate was continuously administered once daily for 28 days in cycle 1, and the tolerance and pharmacokinetic characteristics of multiple doses were evaluated (Figure 2). The patients who experienced DLTs and disease control (complete response [CR], partial response [PR], or stable disease [SD]) at the end of cycle one were permitted to participate in the subsequent 28-days treatment cycles until progressive disease (PD) or intolerance was observed.

We enrolled three to six patients for each dose level, with the goal of having at least three patients complete the cycle 0 and cycle 1 treatments. We made an enrollment regulation for the extra patients (>3) according to the study stage of the last enrolled patient in the present dose level: If the patient had finished cycle 1 day 22, extra screened patients would be enrolled to the next dose level; if not, the current dose level would be selected. If 1/3 of patients experienced a DLT in cycle 0 or cycle 1, expansion to six patients was required for the current dose level. If two or more DLTs were observed in those six patients, dose escalation would be stopped.

DLTs were observed according to the NCI CTCAE version 4.03 and defined as the emergence of any of the following drug-related AEs: hematological toxicity including Grade ≥3 neutropenia or thrombocytopenia and other Grade ≥4 hematological toxicity; nonhematological toxicities including Grade ≥2 atrioventricular block or renal injury; Grade ≥2 nausea, emesis, or diarrhea for ≥7 days despite optimal treatment; Grade ≥3 nausea, emesis, diarrhea, or rash despite supportive care; and alanine aminotransferase (ALT) or aspartate aminotransferase (AST) elevation by more than 5 times the upper limits of normal for ≥7 days and other Grade ≥3 nonhematological toxicities.

The primary endpoints of the study were metrics of safety and tolerance, as determined by the observance of DLTs and MTDs as well as the recommended doses and regimens for subsequent clinical trials of larotinib mesylate. The secondary endpoints included the pharmacokinetic characteristics and preliminary antitumor activity of larotinib mesylate according to RECIST version 1.1. The exploratory endpoints were the abilities of biomarkers to predict the antitumor effect of larotinib mesylate in patients with solid tumors.

### Safety and Tolerance Analyses

Safety was assessed according to the NCI CTCAE version 4.03 including AEs, vital signs, and results from 12-lead echocardiogram, cardiac ultrasound, physical examination, ophthalmic testing, and clinical laboratory tests. Dosage adjustments could be applied from cycle 2 according to the severity of the toxicities. Drug administration would be suspended when drug-related AEs were graded at 3 or 4, and the original dose or reduction dose would be given if the AEs returned to baseline or Grade 1 (nonhematological toxicities)/grade 2 (hematological toxicities) within 14 days.

### Pharmacokinetics Sampling and Analysis

Blood samples (4 ml) for pharmacokinetic analyses were collected in tubes containing K_2_EDTA anticoagulant. The sampling time-points were shown in Figure 2. Blood samples were centrifuged at 3,000 rpm for 10 min at 4°C and stored at –80°C until liquid chromatography-mass spectrophotometry (LC-MS/MS) analysis. Urine and fecal samples were collected only when patients were given the150-mg and 220-mg dose levels at sampling time intervals of 0–4, 4–8, 8–12, 12–24, 24–48, 48–72, 72–96 and 96–120 h in cycle 0.

Plasma pharmacokinetic data (larotinib and major metabolite M5) were analyzed by standard non-compartmental methods using WinNonlin version 7.0 (Certara United States, Inc.) for calculation of pharmacokinetic parameters including peak plasma concentration (C_max_), time to peak plasma concentration (T_max_), area under the plasma concentration–time curve from time 0–24 h (AUC_0-24_), AUC from time 0 to the last measurable concentration timepoint before the next dose (AUC_0-t_), AUC from time 0 to infinity (AUC_0-∞_), terminal elimination half-life (t_1/2_), clearance (CL/F), apparent volume of distribution (V_z_/F) at the first dose, and these parameters at steady state (C_ss,max_, C_ss, min_, T_ss, max_, AUC_ss, 0–24_, AUC_ss, 0-t_, AUC_ss, 0-∞_, CL/F_ss_ and V_z_/F_ss_). Linear mixed effects model was used to explore the dose exposure relationships. Dose proportionality was concluded if the 95% confidence interval (CI) of the linear regression slope was completely contained within the decision interval (1 + ln (0.8)/ln (8)∼1 + ln (1.25)/ln (8)). For urine and fecal data, the accumulative excretion rates of larotinib and M5 were calculated.

### Bioanalysis

Bioanalysis was performed at Frontage Company (Shanghai, China). Validated high-performance LC-MS/MS method was used to quantify the concentrations of larotinib and M5 in plasma, urine and feces samples. Protein precipitation was employed for plasma, urine and feces processing. Plasma larotinib and M5 concentrations were determined using appropriate calibration curves obtained from standards in the range of 0.5–1,000 ng/ml. The interassay precision of the analysis method were 4.2–5.4% for larotinib and 3.9–4.3% for M5. The interassay accuracy of the analysis method were 100.4–103.4% for larotinib and 102.2–102.9% for M5, respectively.

### Tumor Evaluation

Tumor evaluation was performed at screening, the end of cycle 1, and every two cycles of subsequent treatment according to RECIST version 1.1. The disease control rate (DCR) (including CR, PR and SD), objective remission rate (ORR) (including CR and PR), and progression-free survival (PFS) were also evaluated.

### Biomarkers

Blood samples for EGFR mutation site analyses were collected in screening, cycle 1 day 29, cycle 3 days 29 and every 8 weeks for the subsequent follow-up. Second-generation sequencing method was used to detect the amplification and mutation of the related tumor genes.

### Concomitant Medications

Concomitant medications could be given when clinically required. No other anti-tumor therapies were permitted during the study, and drugs that can causea prolonged QT interval were also prohibited. Inhibitors and inducers of CYP3A4 were to be used with caution, because around 85% of larotinib mesylatewas found to be metabolized by CYP3A4 in vitro studies (data not published).

### Statistical Analysis

Statistical analyses were performed using SAS software, version 9.2 (SAS Institute Inc., United States). Descriptive statistics were used to summarize continuous variables as cases, means with standard deviations (SDs), medians, quartiles, maximums and minimums. For categorical variables, frequencies and percentages were used. A linear mixed effects model, relating log-transformed C_max_ and AUC parameters to log-transformed dose, was used to investigate dose proportionality.

## Results

### Demographics and Baseline Data

In total, 74 patients with advanced solid tumors were screened, and 25 were enrolled in the present dose-escalation study. The numbers of enrolled patients for the different dose levels were 3 in the 50–220 mg/d and 400 mg/d dose levels, 6 in the 300 mg/d dose level, and 4 in the 350 mg/d dose level. Among the 25 enrolled patients, the cancer diagnoses included lung cancer in 11 patients; nasopharynx, salivary, and ovarian cancers in 2 patients each; and esophagus, liver, cervical, colon, breast, thymus, esophageal cancer with lung cancer, and bladder cancer with lung cancer in 1 patient each. The majority of patients were male (64%), and the mean (SD) age was 53.5 (8.34) years. The demographic and disease characteristics of the enrolled cases are presented in Table 1.

**TABLE 1:** Summary of baseline demographic characteristics.

Characteristic	Total
Number of patients treated, n	25
Age (y), median (range)	53.0 (36–70)
Gender, n (%)	
Male	16 (64.0%)
Female	9 (36.0%)
BMI (kg/m^2^), mean (SD)	23.57 (3.059)
ECOG PS at screening, n (%)	
0	12 (48.0%)
1	13 (52.0%)
Tumor type, n (%)	
Lung	11 (44.0%)
Ovarian	2 (8.0%)
Nasopharynx	2 (8.0%)
Salivary	2 (8.0%)
Other	8 (32.0%)
All prior therapies, n (%)	
Surgery	11 (44.0%)
Radiotherapy	11 (44.0%)
Chemotherapy	21 (84.0%)
Other antineoplastic therapy	3 (12.0%)

ECOG PS, Eastern Cooperative Oncology Group performance status.

### Treatment Duration

The median (range) treatment durations were 145.0 (85–307) days, 114.0 (29–195) days, 313.0 (29–478) days, 148.0 (148–194) days, 29.0 (29–220) days, 146.0 (1–534) days, and 88.0 (87–146) days, respectively, for the 50-, 100-, 150-, 220-, 300-, 350-, and 400-mg dose levels, respectively. Two patients withdrew from the study before the end of cycle 1; one in 350 mg dose level for Grade 3 diarrhea on the first day of cycle 0 and another in 300 mg dose level due to the occurrence of PD.

### Safety and Tolerability

Overall, larotinib mesylate was well-tolerated at all dose levels, with no DLTs reported. All patients experienced at least one AE, and 24 (96%) experienced at least one drug-related AE. Most drug-related AEs were at Grade 1 or 2, with no Grade 4 or 5 drug-related AEs observed. Five patients (20%) experienced Grade 3 drug-related AEs including decreased lymphocytes (12%, n = 3), diarrhea (4%, n = 1), and nausea (4%, n = 1). Lymphocyte reductions of Grade 3 were observed with the 100-mg and 400-mg dose levels, at approximately 1 week after first dose in two cases and during cycle 5 in another case. No dose adjustment or special treatment was employed. Two of the patients had recovered by subsequent visits, and one withdrew after observation of PD. Grade 3 diarrhea was observed in one patient after the first dose at the 350-mg level. The patient recovered after treatment with intravenous fluids and oral administration of montmorillonite powder and voluntary withdrew from the study. Grade 3 nausea was observed in one patient at the 220-mg dose level after 6 months of larotinib mesylate treatment, and no subsequent observations could be made as the patient was lost to follow-up. Serious AEs (SAEs) occurred in one patient in each of the 220-mg and 350-mg dose levels. One patient experienced a decreased level of consciousness after 6 months of treatment, and the other experienced pulmonary embolism. Both SAEs were considered unrelated to the larotinib mesylate treatment. The most frequently reported drug-related AEs were acne-like rash (68.0%, n = 17), diarrhea (48.0%, n = 12), paronychia (48.0%, n = 12), anemia (48.0%, n = 12), stomatitis (44.0%, n = 11), hand-foot skin reaction (44.0%, n = 11), decreased lymphocyte percentage (40.0%, n = 10), nausea (36.0%, n = 9), hypoalbuminemia (32.0%, n = 8), and weight loss (32.0%, n = 8). Overall, >10% of patients in all treatment cycles experienced drug-related AEs, as summarized in Table 2.

**TABLE 2:** Summary of treatment-related AEs (NCI CTCAE grades) occurring >10% in patients of all groups.

Dose levels	50 mg (N = 3)			100 mg (N = 3)			150 mg (N = 3)			220 mg (N = 3)			300 mg (N = 6)			350 mg (N = 4)			400 mg (N = 3)		
AEs, n	_Grade1_	_Grade2_	_Grade3_	_Grade1_	_Grade2_	_Grade3_	_Grade1_	_Grade2_	_Grade3_	_Grade1_	_Grade2_	_Grade3_	_Grade1_	_Grade2_	_Grade3_	_Grade1_	_Grade2_	_Grade3_	_Grade1_	_Grade2_	_Grade3_
Acne-like rash	0	0	0	0	0	0	2	0	0	2	1	0	6	0	0	1	2	0	3	0	0
Paronychia	0	0	0	0	0	0	0	2	0	1	2	0	2	0	0	2	1	0	1	1	0
Diarrhea	1	0	0	0	0	0	0	0	0	0	0	0	3	2	0	1	1	1	3	0	0
Anemia	0	1	0	1	1	0	2	0	0	1	0	0	2	2	0	1	0	0	1	0	0
Hand-foot skin reaction	0	0	0	0	0	0	0	1	0	0	3	0	2	0	0	2	1	0	1	1	0
Stomatitis	0	0	0	0	0	0	1	0	0	2	0	0	0	4	0	1	1	0	1	1	0
Decreased lymphocyte	0	1	0	0	0	2	1	1	0	0	1	0	0	1	1	0	0	0	0	2	0
Nausea	0	0	0	0	0	0	1	0	0	0	0	1	3	1	0	1	0	0	2	0	0
Hypoalbuminemia	1	0	0	0	0	0	1	0	0	0	1	0	3	1	0	1	0	0	0	0	0
Weight loss	0	0	0	0	0	0	0	0	0	2	0	0	1	2	0	2	0	0	0	1	0
Hematuria	1	0	0	0	0	0	0	0	0	1	0	0	1	1	0	1	0	0	1	0	0
Hyperglycemia	0	0	0	0	0	0	1	0	0	1	0	0	2	0	0	0	0	0	0	1	0
Emesis	0	0	0	0	0	0	1	0	0	0	0	0	2	0	0	0	0	0	1	1	0
Elevated alanine aminotransferase	0	0	0	0	0	0	1	0	0	0	0	0	2	1	0	0	0	0	0	0	0
Decreased total protein	0	0	0	0	0	0	0	0	0	1	0	0	1	1	0	1	0	0	0	0	0
Proteinuria	0	0	0	0	0	0	0	0	0	1	0	0	1	0	0	1	0	0	0	0	0
Xerostomia	0	0	0	0	0	0	0	0	0	0	0	0	3	0	0	0	0	0	0	0	0
Elevated aspartate aminotransferase	0	0	0	0	0	0	1	0	0	0	0	0	2	0	0	0	0	0	0	0	0

NOTE: No treatment-related Grade 4–5 AEs were observed in all dose cohorts during the study.

AE, adverse event; NCI CTCAE, National Cancer Institute Common Terminology Criteria for Adverse Events.

### Pharmacokinetic Properties

**FIGURE 3:**
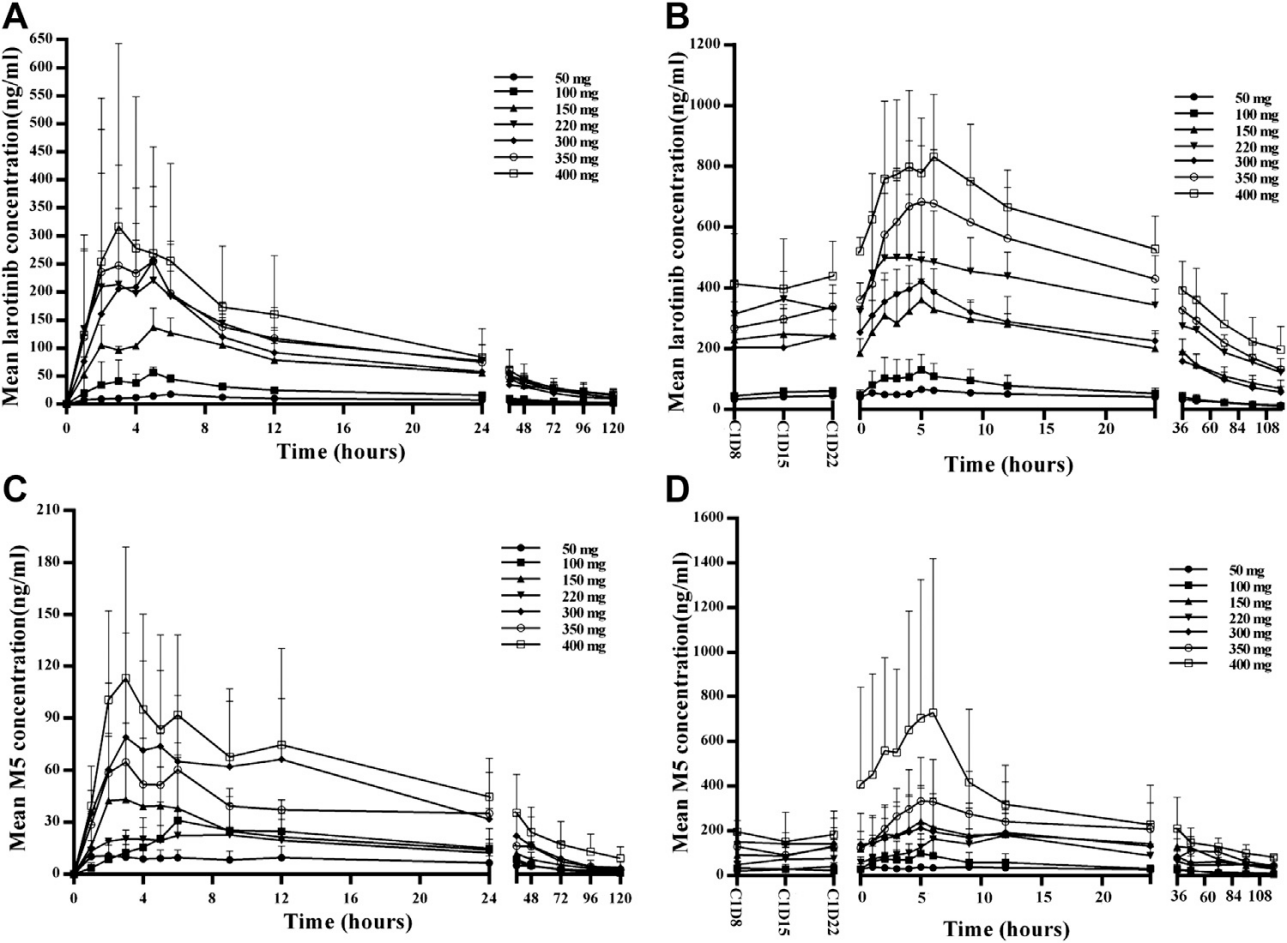
Mean plasma concentration–time profiles for larotinib and M5 after administration of single and multiple doses of 50, 100, 150, 220, 300, 350 and 400 mg larotinib mesylate **(A)** Mean plasma concentration–time profile of larotinib after a single dose; **(B)** mean plasma concentration–time profile of larotinib after multiple doses; **(C)** mean plasma concentration–time profile of M5 after a single dose; **(D)** mean plasma concentration–time profile of M5 after multiple doses.

The mean larotinib plasma concentration–time profiles after the administration of single and multiple doses are shown in Figure 3. After oral administration of a single dose, larotinib was moderately absorbed with a median T_max_ of 3.5–6.0 h. The mean t_1/2_ values were 39.3–68.6 h and did not show a proportional increase with increasing dosages of administration. The correlative analysis of dosages and pharmacokinetic properties showed that the slopes (95% CI) were 1.214 (0.88–1.54), 1.062 (0.73–1.39), and 1.297 (0.95–1.64) for ln AUC_0-24_, ln AUC_0-∞_, and ln C_max_, respectively, showing some overlap but not completely contained in the decision interval (0.89–1.11). Accordingly, no clear linear pharmacokinetic characteristic was observed. At multiple doses were administered, steady-state properties were reached after 8 days of continuous administration. The accumulation factor R1 values (AUC_τ_, cycle 1/AUC_0–24h_, cycle 0) were 5.0, 3.2, 3.3, 3.4, 3.2, 4.6, and 6.3 for the 50–400 mg dose levels, showing obvious accumulation. The plasma pharmacokinetic parameters calculated for single and multiple doses of larotinib are presented in Table 3.

**TABLE 3:** Pharmacokinetic (PK) properties of larotinib after oral administration of single and multiple doses of 50-, 100-, 150-, 220-, 300-, 350- and 400-mg larotinib mesylate tablets.

PK Variables	Dosage (mg)						
	50 mg	100 mg	150 mg	220 mg	300 mg	350 mg	400 mg
Single oral administration No	3	3	3	3	6	3	3
T_max_, median (min–max), h	6.00 (5.00–6.00)	5.00 (3.00–6.00)	5.00 (2.00–5.00)	5.00 (2.00–5.00)	5.00 (2.00–5.00)	3.50 (2.00–5.00)	5.00 (3.00–6.00)
C_max_, mean (min–max), ng/mL	17.42 (15.96–18.70)	64.08 (48.32–84.51)	149.9 (128.4–174.9)	278.0 (169.0–437.0)	267.3 (106.8–401.0)	299.8 (152.1–590.4)	354.3 (76.53–678.8)
AUC_0-24_, mean (min–max), h*ng/mL	244.7 (229.2–254.1)	652.7 (506.0–822.1)	1987 (1,507–2,705)	3,104 (2,384–4,212)	2,690 (1,153–4,397)	3,236 (1741–5,416)	3,973 (1,031–6,848)
AUC_0-t_, mean (min–max), h*ng/mL	634.9 (561.0–691.4)	1,243 (1,009–1,446)	4,781 (3,586–6,799)	6,359 (5,421–7977)	4,885 (2,208–8,615)	6,120 (3,871–9,164)	7435 (2,119–11,572)
AUC_0-∞_, mean (min–max), h*ng/mL	816.6 (701.3–923.0)	1,390 (1,101–1,597)	5,662 (3,938–8,198)	8,093 (6,503–9,065)	5,554 (2,576–9,951)	6,832 (4,777–9,670)	9,088 (2,747–13,379)
T_1/2_, mean (min–max), h	56.71 (56.02–57.42)	43.86 (35.81–52.38)	39.29 (35.34–45.71)	68.20 (44.62–99.19)	46.20 (39.75–52.26)	44.61 (32.56–56.39)	68.60 (58.44–76.26)
Vz/F, mean (min–max), L	5,071 (4,487–5,831)	4,583 (3,919–5,138)	1,651 (971.9–2040)	2,715 (1,562–3,614)	4,345 (1982–8,779)	3,802 (1700–5,960)	7408 (2,521–16,020)
CL/F, mean (min–max), L/h	62.01 (54.17–71.3)	73.81 (62.61–90.83)	29.11 (18.30–38.09)	27.79 (24.27–33.83)	63.76 (30.15–116.4)	55.78 (36.19–73.27)	70.47 (29.90–145.6)
Multiple oral administration No	3	3	3	3	5	3	3
T_max,ss_, median (min–max), h	5.00 (5.00–6.00)	5.00 (5.00–5.00)	5.00 (2.00–5.00)	6.00 (2.00–12.00)	5.00 (1.00–6.00)	5.00 (4.00–6.00)	6.00 (4.00–6.00)
C_max,ss_, mean (min–max), ng/mL	67.50 (65.34–70.90)	131.36 (85.19–185.4)	371.37 (318.6–470.7)	552.6 (405.4–804.4)	437.9 (300.3–550.7)	694.7 (476.3–840.3)	844.8 (637.1–1,087)
AUC_τ,ss_, mean (min–max), h*ng/mL	1,216 (1,029–1,334)	1967 (1,183–2,756)	6,502 (5,277–8,553)	10,303 (8,707–13,353)	7260 (5,889–8,237)	13,155 (9,608–15,933)	16,081 (12,503–19,042)
AUC_0-t,ss_, mean (min–max), h*ng/mL	3,573 (3,009–4,374)	4,479 (2,803–6,271)	18,237 (15,139–23,862)	30,159 (26,097–34,650)	17,925 (13,660–21,285)	35,937 (28,950–44,107)	44,997 (32,935–54,565)
AUC_0-∞,ss_, mean (min–max), h*ng/mL	4,922 (3,947–6,655)	5,196 (3,387–7244)	24,555 (19,828–33,984)	43,641 (36,075–47,906)	22,592 (16,340–28,368)	47,327 (39,584–60,144)	70,416 (50,669–96,144)
T_1/2,ss_, mean (min–max), h	61.20 (50.41–72.73)	44.79 (42.04–49.22)	61.21 (55.71–68.70)	75.66 (66.11–83.41)	54.34 (47.58–67.58)	58.60 (50.04–64.28)	87.05 (66.36–102.4)
V_z_/F_,ss_, mean (min–max), L	921.4 (788.3–1,097)	1,396 (837.2–2097)	563.7 (437.5–646.3)	552.9 (513.1–581.7)	1,061 (904.9–1,260)	640.7 (539.6–784.4)	753.7 (594.3–1,052)
CL/F_,ss_, mean (min–max), L/h	10.73 (7.513–12.58)	21.17 (13.80–29.53)	6.512 (4.414–7.565)	5.126 (4.592–6.098)	13.91 (10.58–18.36)	7.648 (5.819–8.842)	6.087 (4.160–7.894)
R1 (min-max)	4.998 (4.048–5.817)	3.231 (1.439–4.376)	3.302 (3.162–3.502)	3.360 (3.170–3.652)	3.235 (1.713–5.108)	4.639 (2.942–5.519)	6.345 (2.781–12.12)
R2 (min-max)	3.885 (3.711–4.152)	2.308 (1.008–3.837)	2.466 (2.177–2.691)	2.068 (1.841–2.399)	1.888 (1.114–2.813)	2.757 (1.423–3.716)	4.187 (1.602–8.325)

ss = steady state; SD = standard deviation; T_max_ = time to peak plasma concentration; C_max_ = peak plasma concentration; AUC = area under the plasma concentration curve; T_1/2_ = terminal elimination halt-life; V_z_/F = apparent volume of distribution; CL/F = apparent clearance; R1 = AUC_τ,cycle1_/AUC_0–24h,cycle0_; R2 = C_max, cycle1_/C_max, cycle0_.

The mean plasma concentration–time profiles for the major metabolite M5 after administration of single and multiple doses of larotinib are shown in Figure 3, and the corresponding results for M5 plasma pharmacokinetic parameters are presented in Table 4. M5 also showed obvious accumulation with R1 values of 5.08, 3.73, 7.77, 8.14, 3.88, 5.94, and 7.33 for the respective increasing dose levels.

**TABLE 4:** Pharmacokinetic (PK) properties of M5 after oral administration of single and multiple doses of 50-, 100-, 150-, 220-, 300-, 350- and 400-mg larotinib mesylate tablets.

PK Variables	Dosage (mg)						
	50 mg	100 mg	150 mg	220 mg	300 mg	350 mg	400 mg
Single oral administration No	3	3	3	3	6	3	3
T_max_, median (min–max), h	6.00 (1.00–6.00)	6.00 (5.00–12.00)	3.00 (2.00–9.00)	3.00 (2.00–9.00)	6.00 (2.00–12.00)	3.00 (2.00–6.00)	3.00 (2.00–12.00)
C_max_, mean (min–max), ng/mL	11.826 (5.276–20.72)	35.75 (5.310–72.28)	49.09 (8.958–91.24)	28.19 (19.05–42.48)	114.7 (32.69–191.0)	80.07 (67.58–94.76)	120.3 (78.46–200.6)
AUC_0-24_, mean ((min-max)), h*ng/mL	203.8 (113.5–356.8)	474.5 (93.34–887.0)	575.5 (167.6–1,039)	425.5 (205.8–638.6)	1,323 (386.9–2,681)	978.2 (888.5–1,036)	1,646 (1,043–2,450)
AUC_0-t_, mean ((min-max)), h*ng/mL	515.4 (342.1–724.1)	860.4 (223.7–1,612)	1,195 (583.5–1842)	803.7 (476.8–1,096)	2,440 (674.1–4,640)	1986 (1871–2091)	3,612 (1774–4,733)
AUC_0-∞_, mean ((min-max)), h*ng/mL	626.1 (367.5–765.6)	920.8 (256.5–1714)	1,347 (760.0–1916)	949.6 (558.0–1,306)	2,616 (705.5–5,111)	2096 (1980–2,212)	4,284 (1956–5,553)
T_1/2_, mean ((min-max)), h	46.92 (24.98–88.00)	37.21 (33.32–41.31)	41.01 (26.43–59.33)	64.24 (41.98–75.88)	32.64 (23.25–44.17)	28.57 (26.44–32.64)	48.40 (40.13–53.58)
Vz/F, mean ((min-max)), L	5,346 (2,617–8,519)	9,654 (3,477–18,742)	8,598 (2,986–16,894)	22,148 (18,438–24,125)	8,096 (2,580–15,907)	6,925 (6,036–8,322)	7660 (5,352–11,839)
CL/F, mean ((min-max)), L/h	89.47 (65.30–136.0)	191.5 (58.33–389.9)	128.6 (78.30–197.4)	262.0 (168.4–394.3)	178.3 (58.70–425.2)	167.3 (158.2–176.7)	117.1 (72.04–204.5)
Multiple oral administration No	3	3	3	3	5	3	3
T_max,ss_, median (min–max), h	9.00 (1.00–12.00)	5.00 (5.00–24.00)	5.00 (5.00–5.00)	6.00 (6.00–12.00)	6.00 (0.00–12.00)	5.00 (2.00–6.00)	5.00 (3.00–6.00)
C_max,ss_, mean ((min-max)), ng/mL	44.94 (42.31–46.50)	110.3 (46.48–187.0)	242.4 (79.13–365.4)	185.4 (123.2–291.1)	230.9 (41.74–516.1)	353.4 (159.3–503.1)	752.0 (212.5–1,511)
AUC_τ,ss_, mean ((min-max)), h*ng/mL	788.7 (681.9–874.4)	1,356 (539.7–2,272)	4,221 (1,269–6,440)	3,128 (2,117–4,796)	4,092 (701.6–8,440)	5,852 (2,497–10,089)	9,572 (3,610–16,790)
AUC_0-t,ss_, mean ((min-max)), h*ng/mL	2,332 (1863–2,725)	2,737 (1,320–4,450)	12,043 (3,445–17,143	7998 (4,661–13,758)	10,114 (2,229–22,840)	14,576 (11,235–19,076)	22,565 (12,430–32,554)
AUC_0-∞,ss_, mean ((min-max)), h*ng/mL	3,057 (2,170–3,835)	3,077 (1,643–4,952)	13,510 (4,714–19,002)	13,661 (6,569–27,809)	15,385 (2,328–38,578)	16,911 (14,280–20,484)	40,022 (31,831–51,472)
T_1/2,ss_, mean ((min-max)), h	53.79 (35.79–84.19)	47.38 (35.09–65.09)	38.79 (30.91–53.13)	89.40 (49.83–124.7)	70.75 (28.95–95.37)	36.94 (31.47–44.32)	151.3 (38.91–346.4)
V_z_/F_,ss_, mean ((min-max)), L	1,258 (815.5–1,584)	3,011 (1,022–5,716)	1,068 (368.2–2,439)	2,777 (1,423–4,501)	3,861 (1,070–8,728)	1,150 (775.7–1,567)	1912 (610.8–3,883)
CL/F_,ss_, mean ((min-max)), L/h	17.29 (13.04–23.04)	39.66 (20.19–60.87)	16.21 (7.894–31.82)	24.90 (7.911–33.49)	48.76 (7.777–128.8)	21.17 (17.09–24.51)	10.41 (7.771–12.57)
R1 (min-max)	5.08 (1.911–7.136)	3.73 (2.561–5.782)	7.77 (6.197–9.527)	8.140 (4.899–12.01)	3.882 (0.7928–6.478)	5.94 (2.473–9.743)	7.327 (2.499–16.09)
R2 (min-max)	5.048 (2.221–8.020)	4.874 (2.587–8.753)	6.282 (4.005–8.833)	6.549 (5.346–7.447)	2.255 (0.7851–4.025)	4.477 (2.358–5.762)	8.171 (2.597–19.26)

ss, steady state; SD, standard deviation; T_max_, time to peak plasma concentration; C_max_, peak plasma concentration; AUC, area under the plasma concentration curve; T_1/2_, terminal elimination halt-life; V_z_/F, apparent volume of distribution; CL/F, apparent clearance; R1, AUC_τ,cycle1_/AUC_0–24h,cycle0_; R2, C_max, cycle1_/C_max, cycle0_.

In the 150- and 220-mg groups, the 0–120 h excretion recovery percentages of larotinib were 7.4078% (1.3963% in urine, 6.0115% in feces) and 9.2492% (1.3587% in urine, 7.8905% in feces), respectively. For M5, these recovery percentages were 7.3986% (0.0111% in urine, 7.3875% in feces) and 7.1600% (0.0045% in urine, 7.1555% in feces), respectively.

### Antitumor Efficacy

**FIGURE 4:**
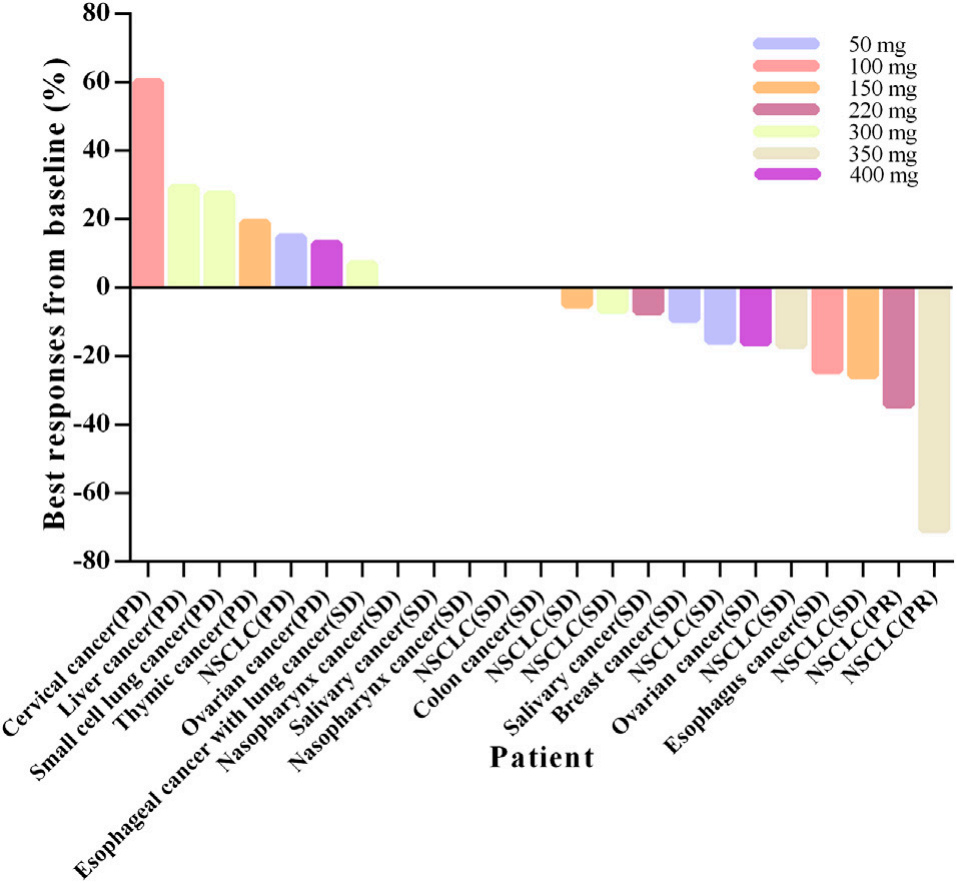
Percentage change in tumor size of the target lesions by RECIST (version 1.1) in patients from baseline to best responses. Abbreviations: NSCLC, non-small cell lung cancer.

Twenty-four cases were evaluable for antitumor efficacy by RECIST version 1.1. Two cases with PR were observed, and both were patients with NSCLC. One was a 70-year-old male in the 220-mg group, who had previously experienced treatment failure with paclitaxel, pemetrexed, and platinum compounds. PR was achieved at the end of cycle one and maintained for six cycles, after which time the patient withdrew due to a drug-unrelated SAE. The EGFR G719A-activating mutation was found in this case at the time of screening, and after larotinib treatment, the mutation was no longer detected. The other case was a 36-year-old male in the 350-mg group. Pemetrexed and *cis*-platinum has been used for prior treatment. PR was maintained for 19 cycles, and no EGFR-sensitive or -resistant mutations were detected during the treatment. SD was observed as the best overall response in 15 patients (60%), and the overall DCR was 70.8% (Table 5). The best responses from baseline in target lesions in all the treatment cycles are shown in Figure 4 and the tumor size-time profiles were shown in Supplementary Figure S1.

**TABLE 5:** Best overall therapeutic responses to larotinib mesylate.

Best overall response, n (%)	Dosage (mg)							
	50 mg (n = 3)	100 mg (n = 3)	150 mg (n = 3)	220 mg (n = 3)	300 mg (n = 6)	350 mg (n = 3)	400 mg (n = 3)	Total (n = 24)
Objective response^a^	0 (0)	0 (0)	0 (0)	1 (33.3)	0 (0)	1 (33.3)	0 (0)	2 (8.3)
Disease control^b^	2 (66.7)	2 (66.7)	2 (66.7)	3 (100)	2 (33.3)	3 (100)	3 (100)	17 (70.8)
Complete response	0 (0)	0 (0)	0 (0)	0 (0)	0 (0)	0 (0)	0 (0)	0 (0)
Partial response	0 (0)	0 (0)	0 (0)	1 (33.3)	0 (0)	1 (33.3)	0 (0)	2 (8.3)
Stable disease	2 (66.7)	2 (66.7)	2 (66.7)	2 (66.7)	2 (33.3)	2 (66.7)	3 (100)	15 (62.5)
Progressive disease	1 (33.3)	1 (33.3)	1 (33.3)	0 (0)	4 (66.7)	0 (0)	0 (0)	7 (29.2)
Not evaluated	0 (0)	0 (0)	0 (0)	0 (0)	0 (0)	0 (0)	0 (0)	0 (0)

^a^ : Includes complete response and partial response.

^b^ : Includes complete response, partial response and stable disease.

### Biomarkers

Mutations and amplifications of tumor genes were detected in peripheral blood samples. The results showed that changes in the abundance of tumor gene mutations in peripheral blood reflected the disease status to a certain extent. *EGFR* mutations were detected at baseline in five patients (003, G719D mutation; 301, G719A mutation; 201, L858 mutation; 202 and 501, deletion in the exon 19). After cycle one of treatment with larotinib mesylate, these mutations were either no longer detected or detected at a lower abundance, and the corresponding efficacy evaluation showed 1 case of PR and 4 cases of SD, which indicated agood inhibitory effect in patients with *EGFR* mutations.

## Discussion

This phase 1 trial was the first-in-human study to evaluate the tolerance, pharmacokinetics, and preliminary efficacy of single- and multi-dose larotinib mesylate in patients with advanced solid tumors. All dose levels administered were well-tolerated with no MTD established. Therefore, in terms of safety, 50–400 mg/d larotinib mesylate via oral administration can be selected for subsequent clinical trials.

The majority of common AEs were EGFR-related skin, mucosal, and gastrointestinal reactions, including acne-like rash, diarrhea, paronychia, stomatitis, hand-foot skin reaction, and nausea, consistent with the common AEs reported for most of the EGFR-TKIs on the market (Yap et al., 2010; Bahleda et al., 2015; Califano et al., 2015; Yang et al., 2017; Kelly, Shepherd, Krivoshik, Jie and Horn, 2019). Most of the AEs were effectively controlled with symptomatic treatments, and no DLT occurred. In general, these AEs presented an obvious dose-dependency, with no or rare occurrence at lower dose levels (50 or 100 mg) and a higher incidence at higher dose levels (150–400 mg). Other drug-related AEs emerged at different dose levels, and no obvious dose-dependent relationship was observed.

The PK analysis of larotinib showed no linear PK characteristic, and this may be attributed to the small sample size and high inter-individual heterogeneity. Double peaks were observed in the plasma concentration–time profiles for both single and multiple doses, which indicated recirculation process taking place. The results can be explained by the fact that larotinib is a substrate of P-glycoprotein and reabsorbed via the P-glycoprotein–mediated larotinib efflux. The drug did show obvious accumulation after the administration of multiple doses, with the R1 value exceeding three at all dose levels. A time-dependent CL/F and V_z_/F were observed between the first dose and the steady-state, because of the increased AUC after multiple dose. The t_1/2_ was comparable between the first dose and steady-state, no obvious time-dependence was observed. The trough larotinib concentrations at steady-state with the lower dose levels (50 and 100 mg) were above the IC_50_ values shown to inhibit EGFR, and with higher dose levels (150–400 mg), these concentrations were above the IC_50_ values to inhibit EGFR, HER2, HER4, PIPK2, IRAK1, BTK, and BLK, suggesting the higher dose levels may offer better and broader therapeutic effects than the lower dose levels. The V_z_/F values were very large for all dose levels, showing the drug was mainly distributed in tissues. The 0–120 h excretion recovery percentages of larotinib and the major metabolite M5 were low, and the main excretion pathway was feces, implying that the drug was slowly eliminated in the tissues and supporting the safety of the drug in patients with chronic renal insufficiency.

The efficacy analysis revealed favourable antitumor activities with two cases of PR and a DCR of 70.8% after larotinib mesylate treatment. All five patients with *EGFR* mutations at baseline achieved PR or SD after larotinib mesylate treatment, which indicated antitumor activity of this agent for the treatment of patients with *EGFR* mutations. Notably, tumors expressing wild-type EGFR are often insensitive to EGFR-TKIs(Mahipal et al., 2014; Ojemuyiwa et al., 2014; Lisberg and Garon, 2017; Matsuda et al., 2017; Thomas et al., 2019), but in the present study, one case with no EGFR-sensitive mutations achieved and maintained a PR for 19 cycles. Considering the lower IC_50_ value calculated in the larotinib mesylate *in vitro* study, this observation suggests larotinib mesylate may be superior to other TKIs for targeting wild-type EGFR tumors.

In conclusion, larotinib mesylate was well-tolerated when orally administered at doses from 50 to 400 mg/d and showed evidence of antitumor activity in patients with advanced solid tumors. Further studies evaluating this drug’s antitumor activity are ongoing in for multiple tumor types with different genotypes.

## Data Availability

The original contributions presented in the study are included in the article/Supplementary Material, further inquiries can be directed to the corresponding author.
